# Drug Lag and Associated Factors for Approved Drugs in Korea Compared with the United States

**DOI:** 10.3390/ijerph19052857

**Published:** 2022-03-01

**Authors:** Inhye Cho, Euna Han

**Affiliations:** 1 Department of Pharmaceutical Medicine and Regulatory Sciences, Yonsei Institute of Pharmaceutical Sciences, College of Medicine and Pharmacy, Yonsei University, Seoul 03722, Korea; inhye.cho@yonsei.ac.kr; 2 Department of Pharmacy, Yonsei Institute of Pharmaceutical Sciences, College of Pharmacy, Yonsei University, Incheon 21983, Korea

**Keywords:** drug lag, drug access, new drug, regulatory approval, Korean Health Authority (MFDS)

## Abstract

(1) Background: Drug lag, the delay between the first global regulatory approval and approval by the national health authorities in other countries, impacts the accessibility of drugs. Although the Korean pharmaceutical market has grown significantly, most of its innovative drugs for public health depend on imports from foreign pharmaceutical markets. (2) Methods: We extracted data from the official websites of the Korean Ministry of Food and Drug Safety (MFDS) and the US Food and Drug Administration. Information on new molecule entity drugs, approved as imported drugs by MFDS from 2000 to 2019, was extracted. Multivariate Cox proportional hazard models on drug approval were estimated. (3) Results: In total, 424 drugs were analyzed. Orphan drugs designated by MFDS were less likely to receive approval (HR = 0.731, 95% CI: 0.572–0.934). The drugs with Korean MAHs were less likely to obtain drug approval than those with MAHs of subsidiaries of multinational pharmaceutical companies (HR = 0.524, 95% CI: 0.371–0.738). In the analyses for non-orphan drugs (*n* = 37), oncology drugs that need local clinical study (HR = 0.247, 95% CI: 0.093–0.657) and drugs that need more patients in a local clinical study (HR = 0.993, 95% CI: 0.988–0.999) were less likely to receive approval, with longer drug lag. The higher number of clinical studies in Korea was associated with a shorter drug lag (HR = 2.133, 95% CI: 1.196–3.805). (4) Conclusions: Our findings imply that Korean pharmaceutical companies should augment their research capabilities for new drug development. Furthermore, consideration of orphan drugs used in rare diseases is needed for drug approval to ensure the availability of these drugs in the market without approval delays.

## 1. Introduction

The pharmaceutical industry is based on high levels of technology and strong regulation by national regulatory authorities [1,2]. Regulatory approval is an important milestone in drug development and commercialization, because drugs can be used and marketed from the date of regulatory approval. Therefore, local regulatory approval is directly related to the accessibility of drugs to patients. Because each regulatory authority approves drugs in the country after review of safety and efficacy in terms of the risk–benefit profile, drug approval duration varies by each country or region [3].

Drug lag is defined as the delay between the global first approval (usually from the competent authority of the United States Food and Drug Administration (FDA) and the European Medicines Agency (EMA)) and the regulatory approval from the national health authority in each country [3]. Drug lag is recognized as an issue [4,5,6,7], given that a few countries, particularly the United States (US), lead the development of innovative drugs, and most countries provide access to such innovative drugs to local patients through local registration and import [8].

Preparing the regulatory pathway of the local new drug application (NDA) requires generating local specific data for the target indication and population to address the potential ethnic difference of the efficacy or safety of the drug [9,10,11,12]. Bridging clinical studies are required to extrapolate the foreign clinical data to local populations for local regulatory approval [13]. Such requirements of local clinical studies have contributed to the drug lag in Asian countries, including Korea, because these bridging studies are usually initiated after the design of international pivotal studies is confirmed. This is to help local studies adopt a similar design of the global pivotal studies for effective comparison of efficacy and safety [14], although multiregional clinical trials (MRCTs) are used for global drug development across different regions simultaneously [15].

Regulatory authorities have introduced the orphan drug designation pathway to promote the research and development (R&D) of drugs, especially for the treatment of rare diseases [16]. If a drug is designated an orphan drug in Korea, bridging the clinical study in the Korean population is exempted along with local quality control tests, and the priority review is conducted during the application for reimbursement after regulatory approval [17]. As these factors influence the decision of pharmaceutical companies to launch their products in a country, the adopted regulatory pathway of each drug for approval is expected to affect the drug lag. Moreover, the review scope and the period of the regulatory pathway would influence the timing of the regulatory approval.

The pharmaceutical industry in Korea ranked 13th in the world in 2016 [18], accounting for 1.8% of the global market in sales [19]. The Korean pharmaceutical market is expected to take a greater share of the global pharmaceutical market in the future, with an annual growth rate of 14.5% in 2012–2017 for chemical drugs and 35.6% in 2013–2017 for biologics [19]. However, it still depends on imports for innovative drugs. Therefore, it is important to facilitate the accessibility of key innovative drugs developed overseas through timely regulatory approval for public health. Indeed, disparities in drug access between regions have been studied for implications in many countries [6,7,20,21,22].

This study aims to investigate the factors associated with the drug lag of new drugs in Korea compared with the US. In an era of differentiated treatment choice for each patient and rapid advancement of standard care, this regional disparity in drug accessibility is an important challenge for all countries. Understanding the impacts of the regulatory pathway and local requirements for NDAs on drug lag is crucial to improving accessibility.

## 2. Materials and Methods

### 2.1. Data Collection

This study extracted data on regulatory information from the official websites of the Korean Ministry of Food and Drug Safety (MFDS) [23] and the US FDA [24]. The six assumptions for selecting the potential variables affecting drug lag were as follows.

First, the drugs with the main indication of oncology could be developed through the rapid expansion of regulatory approvals globally based on highly unmet medical needs. Second, the commercial priority of orphan drug registration is lower than other new drugs due to the low market potential and low prevalence of the disease population. Third, change of the local regulations, such as the expansion of the review scope for orphan drugs in 2015, could increase the review period of orphans. Fourth, the maturity of the pharmaceutical industry of the country could impact the drug lag, and this is impacted by the nationality of the pharmaceutical company. Fifth, the regulatory pathway for orphan drug designation and priority review for the innovative characteristics of the drug and urgent medical need could impact the drug lag. Finally, a local study generating local data conducted in Korea could be a potential contributing factor for the approval of NDAs.

Based on these assumptions, the following information was obtained: the approval date of the MFDS and the FDA, status of orphan drug designation of Korea and the US, regulatory pathway in the US, nationality of the drug developer, Marketing Authorization Holder (MAH) of the drug in Korea, the origin of the drug (chemical or biologicals), Anatomical Therapeutic Code (ATC), approved indication for oncology, number of clinical trials conducted in Korea, number of subjects enrolled in the clinical trials in Korea, number of confirmatory trials (phase III), the period from the Investigational New Drug (IND) (a substance that has been tested in the laboratory and has been approved by the health authority for testing in people. Clinical trials test how well investigational new drugs work and whether they are safe to use; also called an experimental drug, investigational agent, and investigational drug) approval date to the NDA (a document whereby a pharmaceutical manufacturer or its agent requests permission from the health authority for a license to market a drug for one or more specified indications; NDA filed with the FDA as described in 21 C.F.R. § 314, a Biological License Application (BLA) pursuant to 21 C.F.R. § 601.2, or any equivalent or any corresponding application for regulatory approval in any country or regulatory jurisdiction other than the United States)approval date (defined as development lag), and percentage of Korean subjects in the clinical trials of the drug.

The drug developer was considered based on the regulatory information on the respective health authority website and the related articles. The number of patients participating in the clinical studies was assumed based on the applicant’s disclosure of the targeted number of patients.

### 2.2. Study Drugs

Study drugs are approved as imported new molecule entity drugs in Korea. Drugs not approved by the FDA or the MFDS were excluded because the drug lag could not be defined. All new molecule entities approved by the MFDS through a New Drug Application (NDA) from 1 January 2000 to 31 December 2019 were categorized as chemical drugs or biologics. Because the new molecule entity could be approved as an orphan drug or a new drug in the regulatory pathway, this classification was also collected. In the case of several strengths of the product approved with the same regulatory information, the multiple approvals were considered as one.

**Figure 1:**
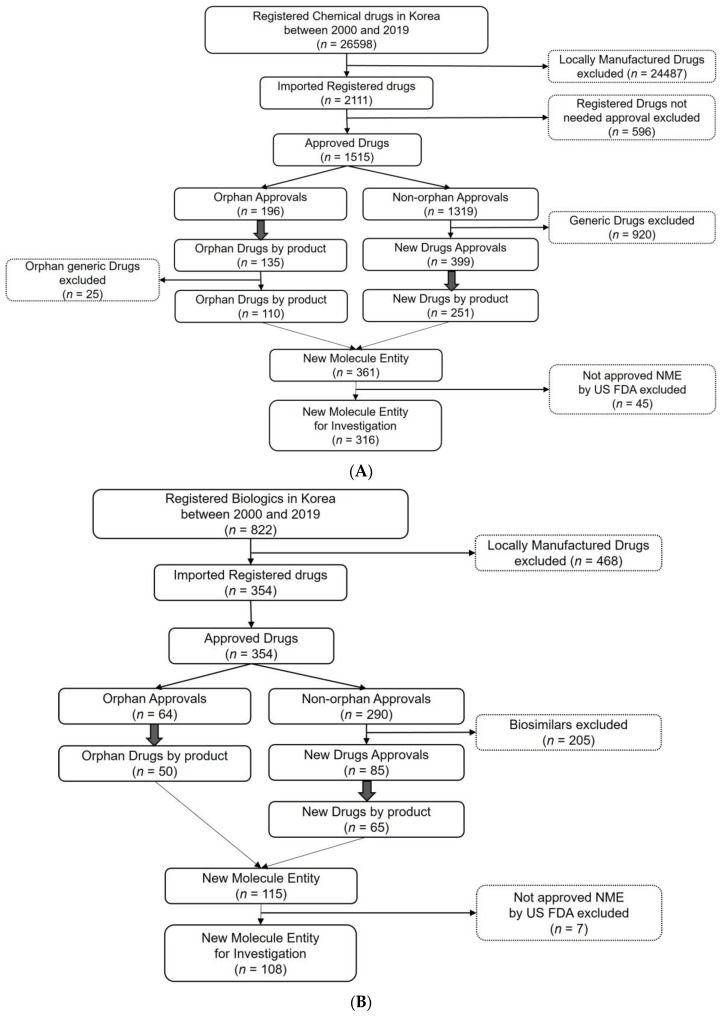
(**A**) Flowchart for the identification of study drugs: Chemical new-molecule entities. (**B**) Flowchart for the identification of study drugs: Biological new-molecule entities.

For chemical drugs, 110 orphan drugs and 251 non-orphan new drugs were approved as new drugs. After excluding the products not approved by the FDA, 316 new chemical entities were investigated. For the biologics, 50 orphan drugs and 65 new drug products were approved under the new marketing authorization. After excluding the products not approved by the FDA, 108 new biological entities were investigated. A total of 424 drugs (316 chemical drugs and 108 biologics) were included as the final sample in this study (Figure 1A,B).

Because information on local clinical trials have been available since 2012, we conducted sub-group analysis for the drugs approved through the new drug pathway since 2012 (*n* = 37).

### 2.3. Variables

#### 2.3.1. Dependent Variable

The study’s dependent variable is the MFDS approval and drug lag period. Drug lag is a continuous variable, which was defined as the number of months delayed from the date of FDA approval in the US to the date of MFDS approval in Korea.

#### 2.3.2. Independent Variables

The following information on the drugs was considered as the potential determinants of the drug lag: the approved year of the drug (as a continuous variable), the status of orphan drug designation of Korea (with new drug review as the reference), the origin of the drug being biological (with chemical as the reference), the regulatory pathway being priority review in the US (with standard review as the reference), the nationality of the drug developer (Europe, Japan, Others, with US as the reference), the MAH of the drug being a Korean company in Korea (with non-Korean company as the reference), the approved drug with oncology indications (with non-oncology drugs as the reference), and being approved since 2015 (with being approved before 2015 as the reference).

For the drugs approved through the new drug pathway since 2012 (*n* = 37), the following information regarding clinical trials for the local studies for the Investigational New Drug (IND) application was additionally considered as determinants for the drug lag: the number of clinical trials conducted in Korea (as a continuous variable) and the number of subjects enrolled in the clinical trials in Korea (as a continuous variable). The requirement of local clinical study only applies to new drugs not designated as orphan drugs.

#### 2.3.3. Statistical Analysis

Univariate regression analysis and the Wilcoxon rank-sum test were performed to identify factors associated with the drug lag. Multivariate linear regression and Cox proportional hazard models were used in a stepwise approach with the addition of the identified potential factors. SAS^®^ Studio Version 3.8 (SAS Institute Inc., Cary, NC, USA) was used for all statistical analyses. The significance level was set at 10%.

This study was reviewed and approved by the Yonsei Institutional Review Board (approval number: 7001988-202008-HR-961-01E).

## 3. Results

The characteristics of the 424 selected drugs are shown in Table 1. The nationalities of the pharmaceutical companies that develop the drugs and commercialize them were 34.2% US (*n* = 145), 55% Europe, including Switzerland (*n* = 233), 8.5% Japan (*n* = 36), and 2.4% others (*n* = 10). Regarding the MAH, 13.4% were Korean companies (*n* = 57) and 86.6% were subsidiaries of multinational pharmaceutical companies (*n* = 367). Among the studied drugs, the proportion of the orphan drugs designated by the MFDS was approximately one-third (34.7%, *n* = 147). Similarly, 25.9% (*n* = 110) were confirmed as orphan drugs designated by the FDA in total studied drugs, substantially overlapping the designation in Korea. The proportion of priority review by the FDA was 40.1% (*n* = 170), and the proportion of drugs designated both for the priority review and orphan drug designation by the FDA was 11.6% (*n* = 49). New molecules developed for oncology drugs were 32.1% (*n* = 136). The results of the classification by ATC code showed a similar proportion of drugs categorized as category L (antineoplastic and immunomodulatory agents), at 33.7%, compared with the group of oncology drugs at 32.1%.

### 3.1. Associations with the Drug Lag and Related Factors

**Figure 2:**
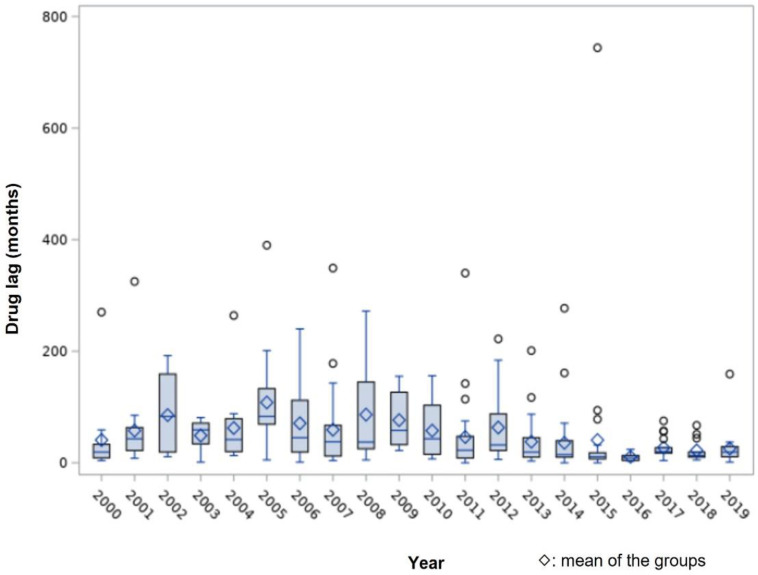
Distribution of drug lag between US and Korea during 2000–2019.

Figure 2 shows the duration of drug lag for the 424 studied drugs for each year. Generally, the delay was up to 50 months, and the drug lag period tended to decrease after 2016; the average duration of drug lag was 40 (2000) to 108 months (2005) before 2016, whereas it was 10.7 months in 2016 and 22 to 27 months after 2016.

**Figure 3:**
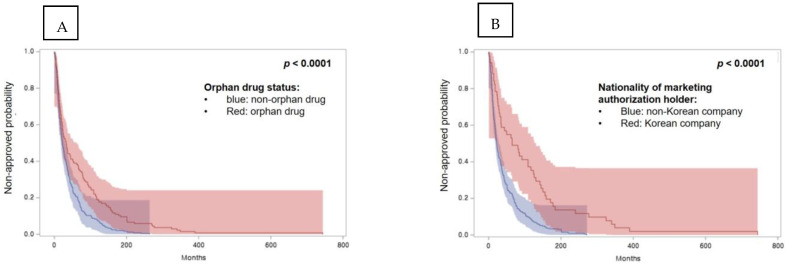

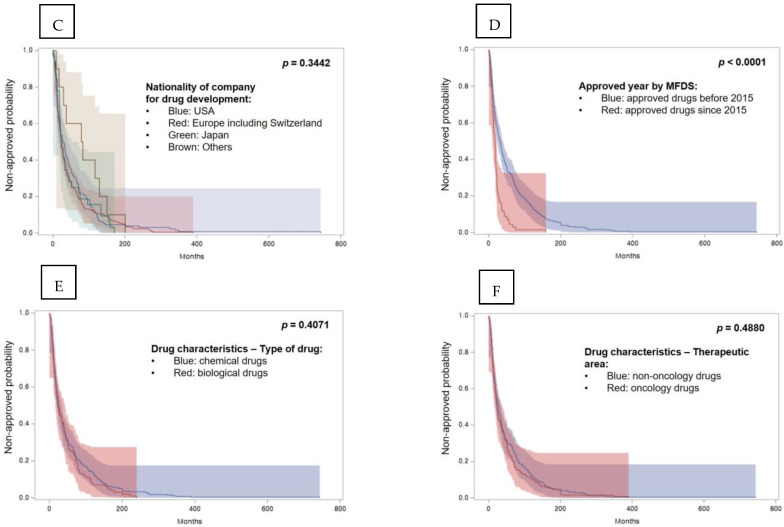
Survival functions for univariate analysis. (**A**) Regulatory pathway in Korea—orphan drug status, (**B**) nationality of the marketing authorization holder—Korean company versus others, (**C**) nationality of pharmaceutical company for drug development, (**D**) approved year by Ministry of Food and Drug Safety since 2015, (**E**) drug characteristics—type of drug: biological drugs vs. chemial drugs, (**F**) drug characteristics—therapeutic area: oncology drugs vs. non-oncology drugs.

The survival functions in univariate analysis are shown in Figure 3. Results show that the time to obtain regulatory approval in Korea for non-orphan drugs and drugs developed by a Korean company was shorter than for the respective counterparts.

Results of multivariate analysis using the Cox proportional hazard model affecting drug lag are shown in Table 2. Orphan drugs designated by the MFDS were 0.731 times less likely to receive approval (HR = 0.731, 95% CI: 0.572–0.934). Drugs with a Korean MAH were 0.524 times less likely to obtain drug approval than those with the MAH of a subsidiary of a multinational pharmaceutical company (HR = 0.524, 95% CI: 0.371–0.738). Moreover, drugs approved after 2015 were 2.02 times more likely to receive drug approval (HR = 2.02, 95% CI: 1.526–2.672).

Furthermore, the nationality of drug developers did not significantly impact drug lag. The status of the US orphan drug designation and priority review, which were considered to have high correlations with the status of the MFDS orphan drug designation, was also not significant. The category of the drugs, chemical drugs or biologics, was also not significant. Finally, the therapeutic area of the drug, categorized as oncology or others, did not show a significant association.

A multivariate linear regression on drug lag duration was conducted to have more intuitive interpretations of the association of the factors with the drug lag (Table S1). The overall results were comparable to the multivariate Cox proportional hazard model. In summary, orphan drugs designated by the MFDS or the MAH of Korean pharmaceutical company showed a statistically significant positive association with the drug lag probability and duration in each analysis. In contrast, drugs approved after 2015 showed a statistically significant negative association with drug lag probability and duration in each estimation.

### 3.2. Association of the Potential Delaying Factors of the Local Study on Drug Lag

To assess the association of the local study on drug lag, variables related to the target enrolment number of patients and the number of local clinical studies were analyzed. The distribution of drug lag for 37 non-orphan drugs is shown in Figure S1. The Wilcoxon rank-sum test showed a significant difference for the drug lag for oncology drugs (Figure S2).

The multivariate Cox proportional hazard model results on drug lag in non-orphan drugs are shown in Table 3. Oncology drugs were 0.247 times less likely to receive approval (HR = 0.247, 95% CI: 0.093–0.657). The number of clinical studies in Korea was associated with a higher likelihood of drug lag (HR = 2.133, 95% CI: 1.196–3.805). The number of Korean patients participating in clinical studies in Korea was negatively associated with drug lag (HR = 0.993, 95% CI: 0.988–0.999). Development lag, defined as the period from the IND approval date to the NDA approval date, was negatively associated with drug lag (HR = 0.97, 95% CI: 0.942–0.999, *p* = 0.0421). Chemical versus biological drugs, the nationality of pharmaceutical companies for drug development, and the US regulatory pathway were not significant variables affecting drug lag. The multivariate linear regression conducted with the same potential variables showed that oncology drugs that need local clinical studies and drugs that need more patients in local clinical studies had a longer drug lag. The higher number of clinical studies in Korea was associated with a shorter drug lag (Table S2).

## 4. Discussion

We investigated drug lag for 424 drugs approved as the new molecule entities in Korea and the US from 2000 to 2019. Our findings show that the drug lag for orphan drugs designated by the MFDS increased by 27%, with statistical significance. The drugs with Korean MAHs have a significantly longer drug lag (48%) than those with the MAHs of subsidiaries of multinational pharmaceutical companies. Moreover, drugs approved after 2015 had a significant decrease in drug lag.

The increased drug lag for the orphan drug status was similarly reported in a previous study in Japan for anticancer drugs [11] A study in Japan showed that the median approval lag of orphan anticancer drugs between Japan and the US was 727.0 days, for which submission lag was the main factor rather than a delay of the review. Although the dates of submission and approval of the application are not disclosed in Korea, the previous findings in Japan imply that both submission and review contributed to the total delay. The submission lag could be due to the lead time for preparing for the regulatory requirements and establishing the marketing strategy. Particularly for orphan drugs, as the lower the prevalence of rare diseases leads to the lower market potential, firm-level decisions for drug development could be a delaying factor in their drug lag. The review delays might be attributed to changes in the review scope of the orphan drugs and a consequent increase in the official review period of these drugs by the health authority. However, our findings showed no statistically significant drug lag after 2015, when the review scope changed, implying no major role of the review side in the drug lag.

In the present study, 34.7% of the new-molecule entities were approved through the orphan drug review pathway in Korea. To promote the R&D of the drugs, especially for the treatment of rare diseases, regulatory authorities have introduced the orphan drug designation pathway [16]. The regulatory procedure of orphan drug designation and approval was initiated in 1983 in the US, 2000 in the EU, and 1993 in Japan [16]. Although the criteria for the designation of orphan drugs differ in each country, the benefits usually include preferential tax treatment, lower fees for regulatory review, priority review by the regulatory authority, and the extension of market exclusivity, which are expected to contribute to R&D and commercialization of the orphan drugs [25].

South Korea has relevant regulations for orphan drug designation with two criteria: one for a prevalent population of the disease of less than 20,000 patients in Korea, and the other for the drugs for diseases with no other treatment options or with significantly improved safety and efficacy as compared to alternative treatments in Korea [17]. If a drug is designated an orphan drug in Korea, bridging the clinical study in the Korean population is exempted, along with the local quality-control tests and the priority review during the application for reimbursement after regulatory approval. However, our findings of the delayed drug approval for orphan drugs in Korea suggest that the benefits of orphan drug designation to encourage drug development have limited impact.

This study showed that the drug lag significantly delayed (48%) the drug development of Korean pharmaceutical companies compared with multinational companies, possibly due to the lack of experience of Korean pharmaceutical companies [26]. The US companies have developed one-third of the innovative medicines (36.4%), followed by the United Kingdom (10.4%) and Japan (8.1%) [8]. In the registration process of each drug, the drug’s market potential may be one primary reason multinational companies decide whether to obtain regulatory approval through a subsidiary on their own or through licensing out to a foreign company [27]. After the release of the ICH E17 guideline [15] for multiregional clinical trials (MRCTs) in 2017 to pursue global drug development across different regions simultaneously, active adaptation and implementation of MRCTs was expected to contribute to the reduction of drug lag periods and increase prompt access to drugs, especially in countries that have local bridging data requirements. It is believed that the ICH participation of the MFDS and the introduction of the ICH standard in Korea would decrease drug lag. Our study showed that the drug lag period decreased with statistical significance for drugs approved in 2015 or later, which is possibly an effect of the participation of the MFDS in the ICH in 2016 [28]. Considering that most of the new drugs (86.6% of the total new molecules approved) were developed in foreign countries with advanced pharmaceutical industries in line with the ICH guidelines, the ICH participation might contribute to reduced drug lag via the harmonization of the regulation of different countries and reduction of the regulatory barriers of different requirements [10,29].

Orphan drug designation in the US was initiated in 1983 to stimulate the development of new therapies for rare diseases [30]. The criteria for orphan drug designation have evolved; however, the key criteria are for prevalence and the medical plausibility over alternative treatments as well as the unmet needs of the patients [30,31]. Several previous studies have evaluated the impact of whether drugs were was approved as an orphan drug in the US and the regulatory pathway, including priority review, accelerated approval, and breakthrough therapy [3,12]. Our findings show that the regulatory pathway in the US did not have a significant effect on drug lag. However, we found that orphan drugs approved by the MFDS were subject to delayed approval, implying the background of the regulations for promoting the development of orphan drugs is not successful in Korea in terms of innovation, urgent medical need, and priority review.

The nationality of the drug developers was not statistically significant. Although there is a confirmed, considerable drug lag from approval in the US and the EU to approval in Japan, significant differences between the US and EU were not observed [6,9,32]. Considering that the potential differences between the US and EU in drug development are not significant, the results of this study—that the nationality of the drug developer is not a significant factor affecting the drug lag in Korea—are plausible.

Several previous studies in Japan showed that the requirement of local clinical studies appears to be significantly associated with the longer drug lag as compared with global clinical studies [33,34,35]. Global drug development based on MRCTs is the preferred strategy to avoid redundant clinical studies and improve the overall study design [15]. Whereas the requirement of local clinical development in Korea was identified as a critical factor affecting drug lag in previous studies [10], participation in global trials rather than separate local studies has increased in Korea [36]. Similar to our study, clinical data generation and review issues were reported in Japan and China [32,37].

Oncology drugs had a longer drug lag than drugs for other therapeutic areas in this study. The potential hurdles in oncology drug development include difficulties of clinical study design for relatively small target patient groups. Recent advances in oncology drug development have focused on targeted anticancer therapies with more specified patient populations [38], which would possibly challenge the enrolment of target patients as compared to studies of non-oncology drugs. Moreover, the small number of Korean patients could be a challenge during the review of the MFDS, as hundreds of patients are required to ensure variations in ethnicity; this could impact drug lag in Korea. Difficulties in enrolling ethnic minority patients with cancer are also reported as a usual challenge in the clinical development of oncology drugs [39]. As the scientific review on the methodological and clinical perspectives could vary depending on the regulatory authority, the global drug development of oncology drugs has additional barriers to non-oncology drugs [40], particularly given the limited globally harmonized regulations for oncology drugs [40,41,42]. As more clinical evidence is obtained, efficient construction of the application data package may have a positive impact on the drug review and lead to shorter drug lag. However, our findings of a longer drug lag for a higher number of targeted patients participating in clinical trials in Korea imply that the targeted number of patients may cause the drug’s regulatory review [43].

Some limitations of this study are acknowledged. First, information on the clinical trials in Korea has been disclosed only since 2012, reducing the number of observations. Although the inclusion of the duration for clinical trials in the drug lag calculation is not consistent in previous studies [33,34], the initiation of a clinical study is the first notable milestone of the drug development. Nakajima et al. showed the importance of reducing approval lag with shortened drug development time in Japan [34]. Once more data are collected in a future study on the expanded disclosure of the information for IND in Korea, more meaningful analyses and interpretations can be obtained. Second, several assumptions were made during the data collection for the use of available data. The drug developer was manually identified as part of the overall regulatory information on the health authority website and the related articles. Third, the number of patients participated in the clinical studies was measured with the disclosed information of the targeted number of patients by the applicant. Future studies need to assess the status of the MRCT participation for IND drugs and its impact on the drug lag. The assessment of study qualities of local clinical trials compared with the MRCTs is also needed in the future studies.

This study is the first comprehensive assessment of drug lag and its influencing factors in Korea in the last 20 years. Despite the significant growth of pharmaceutical industries and development of regulatory environments, we confirmed that drug lag other than in major pharmaceutical-developing countries still exists, due mostly to the impacts of local regulatory requirements.

## 5. Conclusions

Our findings of the significant drug lag for orphan drugs and drugs licensed by Korean pharmaceutical companies imply that improved regulatory processes are needed to improve the accessibility of innovative drugs and minimize drug lag in Korea. Furthermore, the urgency of the medical needs of orphan drugs used in rare diseases must also be considered until sufficient new drug development research capabilities are formed for Korean pharmaceutical companies. In the era of the globalization of drug development, encouraging MRCTs and harmonizing regulatory standards and regulatory processes may enhance the accessibility of innovative drugs to patients and thus improve public health.

## Figures and Tables

**Table 1:** Sample characteristics.

Category	*n*	%
**Total drugs approved during 2000–2019**		
Total drugs investigated	424	100
**Category**		
Chemical drugs	316	74.5
Biologics	108	25.5
**Nationality of pharmaceutical company for drug development**		
US	145	34.2
Europe (including Switzerland)	233	55.0
Japan	36	8.5
Others	10	2.4
**Nationality of Marketing Authorization Holder** (**MAH**)		
Korea pharmaceutical company	57	13.4
Multinational pharmaceutical company	367	86.6
**Orphan Drug Designation Status** (**by Ministry of Food and Drug Safety, Korean Health Authority**)		
Yes	147	34.7
No	277	65.3
**Regulatory pathway in US**		
Orphan drug designation	110	25.9
Priority review	170	40.1
Orphan drug designation and priority review	49	11.6
**Oncology drugs**		
Yes	136	32.1
No	288	67.9
**Therapeutic indication** (**Anatomical Therapeutic Classification code**)		
A (Alimentary tract and metabolism)	50	11.8
B (Blood and blood forming organs)	27	6.4
C (Cardiovascular system)	16	3.8
D (Dermatology)	5	1.2
G (Genito-urinary system and sex hormones)	15	3.5
H (Systemic hormonal preparations, excluding sex hormones)	10	2.4
J (Anti-infectives for systemic use)	68	16.0
L (Antineoplastic and immunomodulatory agents)	143	33.7
M (Musculoskeletal system)	6	1.4
N (Nervous system)	29	6.8
P (Antiparasitic products, insecticides, and repellents)	1	0.2
R (Respiratory system)	18	4.2
S (Sensory organs)	14	3.3
V (Various)	22	5.2

**Table 2:** Multivariate analysis using Cox proportional hazard model on drug lag.

Variable (*n* = 424)	Hazard Ratio (95% Confidence Interval)	*p*-Value
**Regulatory pathway in Korea**		
New drug review by Ministry of Food and Drug Safety	Reference	
Orphan drug review by Ministry of Food and Drug Safety	0.731 (0.572–0.934)	0.0121
**Nationality of the Marketing authorization holder** (**MAH**)		
Non-Korean Company	Reference	
Korean Company	0.524 (0.371–0.738)	0.0002
**Drug characteristics** **—Type of drug**		
Chemical Drugs	Reference	
Biological Drugs	1.068 (0.816–1.397)	0.6314
**Drug characteristics** **—Therapeutic area**		
Non-oncology Drugs	Reference	
Oncology Drugs	1.055 (0.848–1.313)	0.6311
**Nationality of pharmaceutical company for drug development**		
US	Reference	
Europe (including Switzerland)	1.031 (0.824–1.291)	0.7883
Japan	0.858 (0.576–1.276)	0.4486
Others	0.824 (0.423–1.603)	0.5682
**Regulatory pathway in US**		
Standard review by US Food and Drug Administration	Reference	
Priority review by US Food and Drug Administration	0.938 (0.736–1.195)	0.6032
**Approval year by Ministry of Food and Drug Safety**		
Approved drugs before 2015	Reference	
Approved drugs since 2015	2.02 (1.526–2.672)	<0.0001
R^2^	0.1542	

**Table 3:** Multivariate analysis using Cox proportional hazard model on drug lag for non-orphan drugs related with clinical studies in Korea.

Variable (*n* = 37)	Hazard Ratio (95% Confidence Interval)	*p*-Value
**Development lag from the initial clinical study to approval date of the drug**	0.97 (0.942–0.999)	0.0421
**Drug characteristics** **—Type of drug**		
Chemical Drugs	Reference	
Biological Drugs	0.87 (0.29–2.605)	0.8029
**Drug characteristics** **—Therapeutic area**		
Non-oncology Drugs	Reference	
Oncology Drugs	0.247 (0.093–0.657)	0.0051
**Nationality of pharmaceutical company for drug development**		
US	Reference	
Europe (including Switzerland)	1.62 (0.643–4.081)	0.3059
Japan	1.162 (0.229–5.895)	0.8566
**Regulatory pathway in US**		
Standard review by US Food and Drug Administration	Reference	
Priority review by US Food and Drug Administration	0.921 (0.317–2.674)	0.88
**Number of clinical studies in Korea**	2.133 (1.196–3.805)	0.0103
**Number of Korean patients participating in clinical studies in Korea**	0.993 (0.988–0.999)	0.0172
**R^2^**	0.3702	

## Data Availability

This study extracted data on regulatory information from the official websites of the Korean Ministry of Food and Drug Safety (MFDS) [23] and the US FDA [24].

## Supplementary Materials

The following supporting information can be downloaded at: https://www.mdpi.com/article/10.3390/ijerph19052857/s1, Figure S1: Distribution of drug lag for new drug approved with local clinical studies between USA and Korea during 2013–2019; Figure S2: Relationship between drug lag in Korea and clinical study related factors; Table S1: Multivariate linear regression analysis for drug lag; Table S2: Multivariate linear regression analysis on drug lag for non-orphan drugs related with clinical studies in Korea.
