# Supplementary material for: Drug Lag and Associated Factors for Approved Drugs in Korea Compared with the United States
Source: Int J Environ Res Public Health. 2022 Mar 1;19(5):2857. doi: 10.3390/ijerph19052857 (PMC8910054; doi:10.3390/ijerph19052857)

Figure S1. Distribution of drug lag for new drug approved with local clinical studies between USA and Korea during 2013-2019.

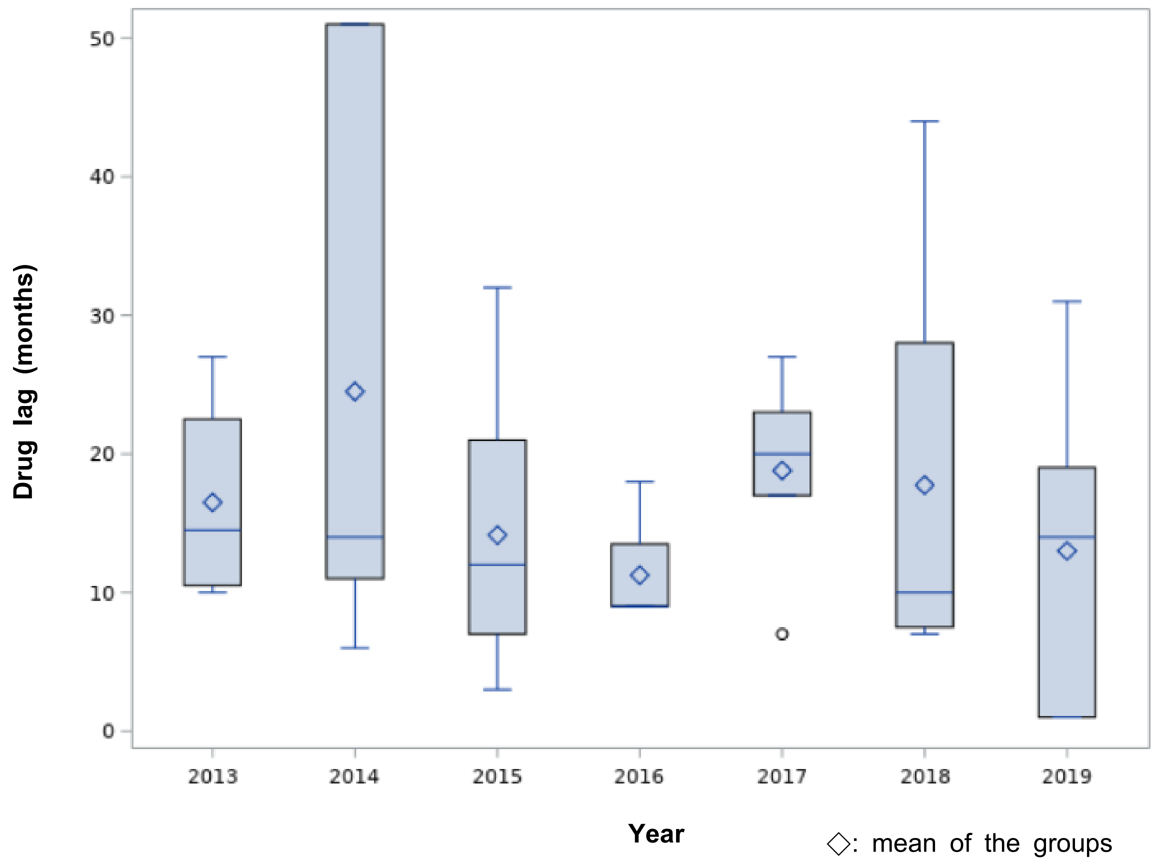

Figure S2. Relationship between drug lag in Korea and clinical study related factors.

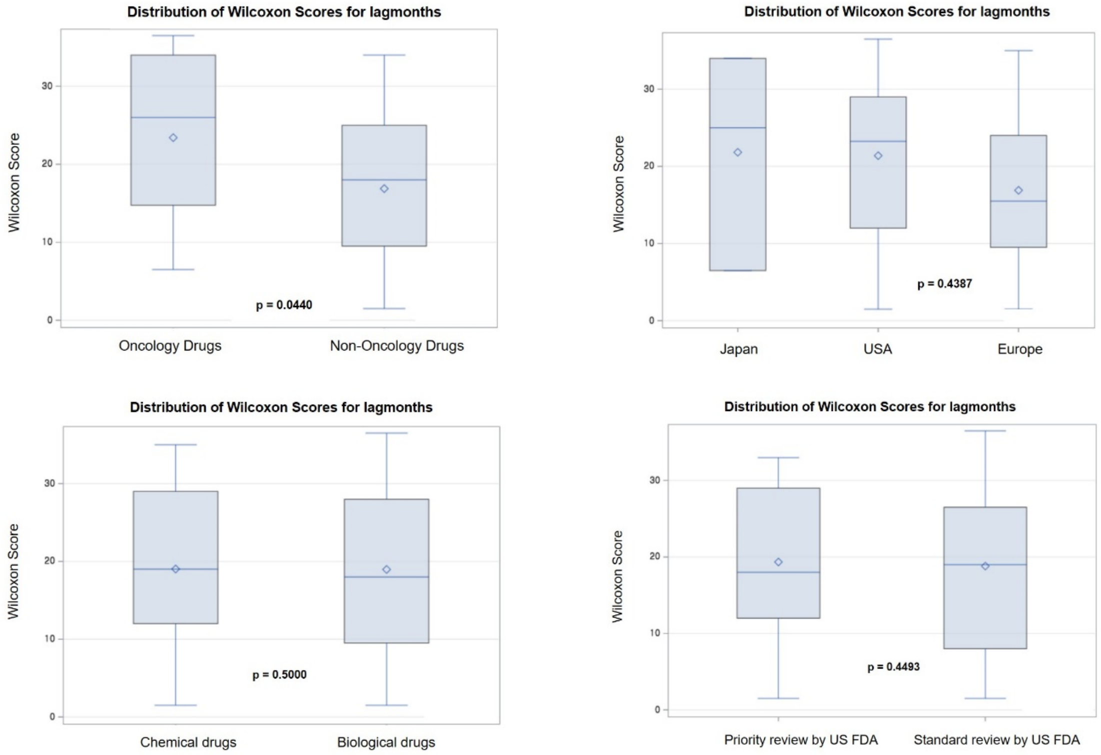

Supplement: Supplementary file 1 [file ijerph-19-02857-s001.zip › supplementary figures.pdf]
