# Supplementary material for: Drug Lag and Associated Factors for Approved Drugs in Korea Compared with the United States
Source: Int J Environ Res Public Health. 2022 Mar 1;19(5):2857. doi: 10.3390/ijerph19052857 (PMC8910054; doi:10.3390/ijerph19052857)
Supplement: Supplementary file 1 [file ijerph-19-02857-s001.zip › supplementary tables.pdf]

Table S1. Multivariate linear regression analysis for drug lag.

Variable		Coefficient	Standard Error	Confidence interval	*p-value*
**Regulatory pathway in Korea**	New drug review by MFDS	reference			
	Orphan drug review by MFDS	25.012	7.4515	(10.4071 - 39.6167)	*0.0008*
**Nationality of the Marketing authorization holder (MAH)**	Non-Korean Company	reference			
	Korean Company	56.526	10.4251	(36.0929 - 76.9585)	*<.0001*
**Drug characteristics - Type of drug**	Chemical Drugs	reference			
	Biological Drugs	-2.126	8.84	(-19.4516 - 15.2005)	0.81
**Drug characteristics - Therapeutic area**	non-oncology Drugs	reference			
	Oncology Drugs	-6.583	7.2312	(-20.756 - 7.5898)	0.3626
**Nationality of pharmaceutical company for drug development**	USA	reference			
	Europe (including Switzerland)	-4.974	7.3202	(-19.321 - 9.3737)	0.4969
	Japan	-2.235	12.7845	(-27.2919 - 22.8225)	0.8612
	Others	-1.483	21.5827	(-43.7841 - 40.8185)	0.9452
**Regulatory pathway in US**	Standard review by US FDA	reference			
	Priority review by US FDA	11.336	7.955	(-4.2551 - 26.9279)	0.1541
**Approved year by MFDS**	Approved drugs before 2015	reference			
	Approved Drugs since 2015	-32.207	8.7913	(-49.438 - -14.9767)	*0.0002*

Table S2. Multivariate linear regression analysis on drug lag for non-orphan drugs related with clinical studies in Korea.

Variable		Coefficient	Standard Error	Confidence interval	*p-value*
**Development lag from the initial clinical study to approval of the drug**		0.181	0.1196	(-0.0532 - 0.4156)	0.1298
**Drug characteristics - Type of drug**	Chemical Drugs	reference			
	Biological Drugs	-3.960	4.4894	(-12.7592 - 4.8391)	0.3777
**Drug characteristics - Therapeutic area**	non-oncology Drugs	reference			
	Oncology Drugs	14.958	3.7716	(7.5653 - 22.3496)	*<.0001*
**Nationality of pharmaceutical company for drug development**	USA	reference			
	Europe (including Switzerland)	-4.391	4.0055	(-12.242 - 3.4593)	0.2729
	Japan	-9.404	7.0536	(-23.2286 - 4.4209)	0.1825
**Regulatory pathway in US**	Standard review by US FDA	reference			
	Priority review by US FDA	-5.046	4.4423	(-13.7528 - 3.6605)	0.256
**Number of clinical studies in Korea**		-6.429	2.3759	(-11.0856 - -1.7724)	*0.0068*
**Number of Korean patients participated in clinical studies in Korea**		0.067	0.0253	(0.0172 - 0.1162)	*0.0083*
